# Medications and Supplements Prescription Patterns during COVID 19 Pandemic in Yemen: A Questionnaire-Based Study

**DOI:** 10.26502/fjhs.079

**Published:** 2022-09-05

**Authors:** Abdullah Chahin, Ghulam Dhabaan, Abdulrahman Buhaish, Mahmoud Shorman

**Affiliations:** 1 Division of Infectious Disease, Loyola University Medical Center and the Stitch School of Medicine at Loyola University, Maywood, Illinois, USA; 2 Department of Microbiology, Mount Sinai Hospital/University Health Network, University of Toronto, Toronto, oN, Canada; 3 Independent Researcher, Sana’a, Yemen; 4 Division of Infectious Diseases, University of Tennessee Medical Center, Knoxville, TN, United States

**Keywords:** COVID-19, Yemen, Coronavirus, Covid-19, pandemic, treatment, supplement, prescription patterns

## Abstract

**Introduction:**

the study aims to better understand the COVID-19 prescription treatments and over the counter regimens in Yemen in view of limited published data and limited availability of COVID-19 testing.

**Methods:**

A 34 question web-based survey was distributed on social media outlets targeting people in Yemen. Data aggregation, analysis, and visualization were performed using Tableau and Microsoft Excel.

**Results:**

2341 individuals reported symptoms concerning for COVID-19 infection, with 25.4% reporting a chronic medical condition. Female patients were less likely to receive medications for treatment in all age groups examined. Azithromycin was the most prescription medication prescribed (32.8%) and vitamin C being the most supplement used (62%). Around 5.5% were on Hydroxychloroquine prophylaxis prior to their diagnosis and only 12.9% of them continued using after diagnosis.

**Conclusions:**

This study provides some important information about the commonly observed treatments and prescription patterns during the COVID-19 pandemic in Yemen during May- July of 2020. The study reflects the influence of global trends in medication prescription even in resource-limited countries.

## 1. Introduction

Coronavirus disease (COVID-19) since it was first reported in December 2019, continues to present a global challenge especially with the rapidly increasing numbers of new cases worldwide, and the lack of definitive treatment [1],[2]. Globally, as of 18 January 2021, 93,805,612 confirmed cases of COVID-19 have been reported to the World Health Organization (WHO) including 2,026,093 deaths [3]. Due to the lack of cure, the management of COVID-19 infected patients continues to focus on supportive care provisions, including, maintaining oxygenation, ventilation, and proper fluid management [4]. The Infectious Diseases Society of America Guidelines on the Treatment and Management of Patients with COVID-19 recommends different options depending on clinical presentation and site of treatment including antiviral therapy, immunotherapy, and cellular therapy. However, these practice guidelines need to be updated frequently as most of these treatment options continue to be under investigation resulting in rapidly emerging published literature [5]. There are different treatment guidelines for COVID-19 worldwide depending on resources [1]. In this paper we report the results of an electronic questionnaire that was developed and distributed online among Yemeni people. We aim to better understand the COVID-19 treatment and over the counter regimens in Yemen in view of limited published data and limited availability of COVID-19 testing.

## 2. Methods

A self-administered, web-based questionnaire - with 34 questions and 56 data points - was designed using google forms. The questionnaire form was distributed to the public using social media outlets. No selection was made to the participants, aside from being In Yemen or from Yemen. Response from 4059 participants Respondents indicated what medical problems they have at baseline by answering a set of yes/no questions and wre able to add additional problem lists as free text. They also were specifically asked about the severity indicators of their illness and had to respond with yes/no. The prescription medications were captured as check boxes for common regimens that the respondents can use. The questionnaire specifically asked about Hydroxychloroquine prescribing before and after the diagnosis. In addition, the respondents can enter the medications as free text. The data was cleaned, and some collected variables were transformed using OpenRefine and python. Data aggregation, analysis, and visualization were performed using Tableau and Microsoft Excel.

## 3. Results

The questionnaire captured 2341 individuals reported symptoms concerning for COVID-19 infection between May and July of 2020. In table 1, we list some of the major characteristics of the population we surveyed. The most prevalent age group was 21–30 years old and 51.7% were male. A sum of 595 individuals reported having a chronic medical illness (25.4%). Obesity was the most reported chronic medical condition (233 respondents, 10%), followed by hypertension (7.7%) and diabetes (6.5%). Asthma was reported as a chronic illness by 54 (2.3%) respondents. As for substance use, we observed that 21.9% of the respondents reported tobacco usage, compared to 57.5% Gat usage.

**Graph 1:**
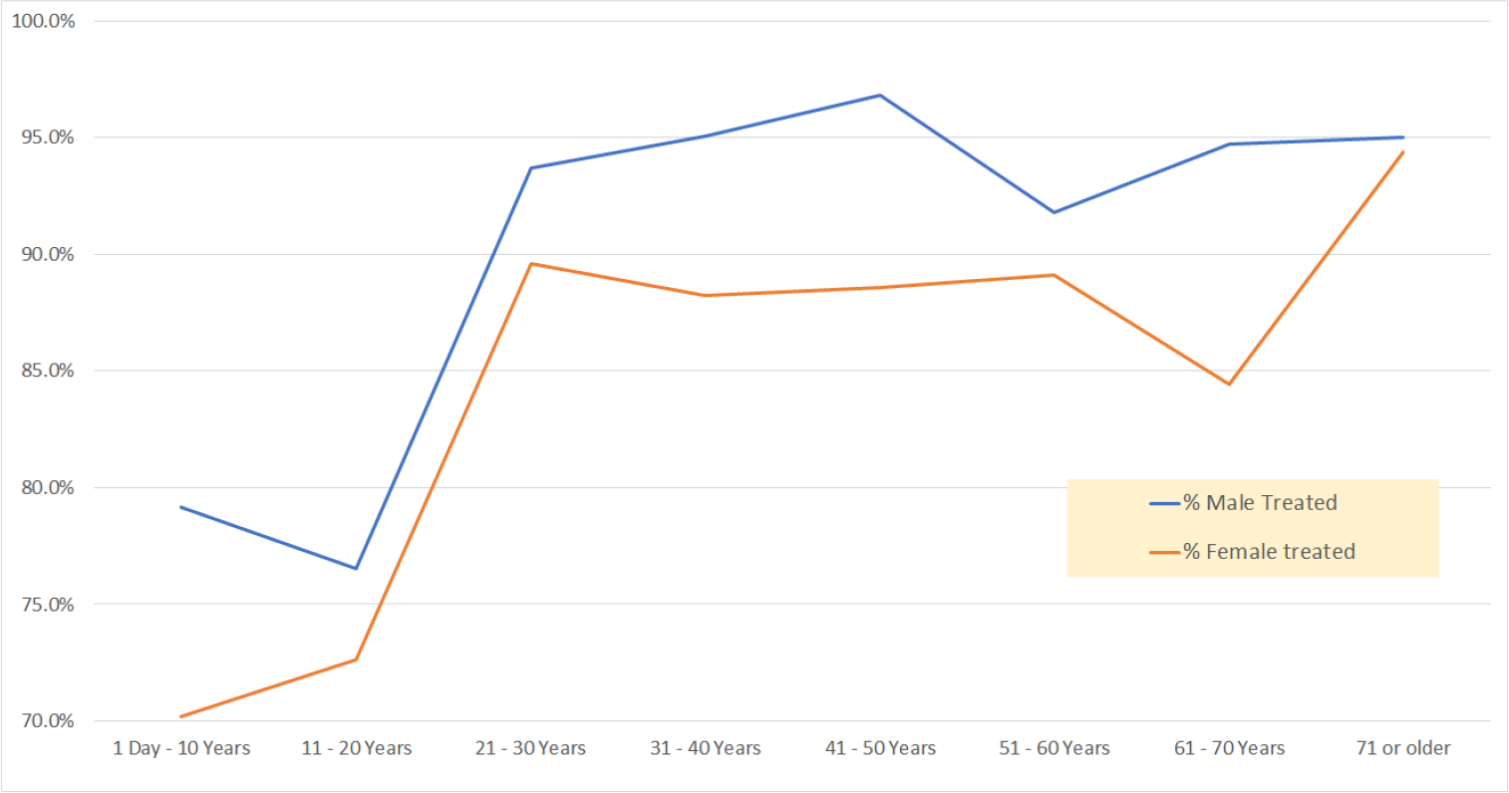
Percentage of male patients receiving treatment vs female across age groups

We began our analysis by looking at the medication prescription patterns by age group and gender. Table 2 demonstrates the percentage of male patients receiving treatment was higher than females. It also shows the progressive tendency to receive treatment with increasing age (from 74.4% in children 10 years of age and younger to 94.7% for elderly age 71 and higher). The prescription percentage is certainly high, but the list of medications received includes many of the over-the-counter medications such as paracetamol (acetaminophen), vitamins, and zinc. When we look at the discrepancy between medication prescription in age, graph 1 shows that this discrepancy is seen among all age groups and it is most pronounced in early adulthood and middle age groups. The difference hovered around 7–10 percentage points. The widest gap of prescription intake between males and females was seen in the 61 – 70 Years age group (94.7% for males vs 84.4% for females).

In table 3, we took a deeper look at the location of our respondents. We also calculated the ratio of the respondents to the sample. We also calculated the ratio of our respondents to population of the area they live in. Not surprisingly, the vast majority of respondents were from the capital municipality (52.6%). The residents of the capital were highly represented (114.83 per 100,000 compared to the average of 8.19 per 100,000 for the rest of the population). This reflects the internet connectivity in a conflict-stricken area. The second most represented area was the city of Ibb (8.89%).

There were more than 30 different substances consumed therapeutically in our questionnaire (table 4). They ranged from antibiotics and systemic steroids to vitamins and herbal remedies. We chose to focus on 7 treatment agents, which have had some data on their use as part of treatment for COVID-19. Those were: Hydroxychloroquine, Azithromycin, Vitamin C, Vitamin D, Zinc, Paracetamol, and systemic Corticosteroids.

We wanted to look at prescription patterns among folks who were not hospitalized, in comparison to those who were admitted to the hospital and those who developed respiratory failure requiring Oxygen supplementation or mechanical intubation. Table 5 shows what percentage of patients received each of these treatments in the 3 clinical categories. Vitamin C was the most prescribed treatment in our study and ranged between 62% for non-hospitalized patients to 74% for hospitalized patients. Azithromycin was the second most commonly used agent, with a significant increase of percentage receiving the treatment with increasing severity (32.1% for non-hospitalized, 57.1% for hospitalized, and 67.5% for patients with respiratory failure). Surprisingly, systemic corticosteroids and paracetamol were the least prescribed treatments, with only 192 individuals (8.2%) receiving paracetamol and only 19 individuals (0.8%) receiving systemic corticosteroids.

As for antibiotics other than hydroxychloroquine and azithromycin, the rate of prescription was surprisingly low. Only 31 participants (1.4%) who were not hospitalized received an antibiotic, 3 of the hospitalized patients, and 2 of the patients who developed respiratory failure (2.1%). Except for paracetamol, all treatments were more commonly prescribed for hospitalized patients and patients with respiratory failure, compared to those who were not hospitalized. The likelihood of receiving those medications seemed to increase with increasing severity in general.

We looked at the frequency of Hydroxychloroquine use among our sample and how many of them continued using it after diagnosis. We also were able to report on how many were started on Hydroxychloroquine after diagnosis. Table 6 shows that 128 individuals (5.5%) were on Hydroxychloroquine prophylaxis prior to their diagnosis and only 18 of those patients (12.9%) continued using the agent. On the other hand, we found that 122 patients (5.2% of the study participants) were started on Hydroxychloroquine treatment after diagnosis. In total, 140 individuals reported receiving Hydroxychloroquine treatment for their COVID-19 infection (6% of the participants).

Finally, we looked at the most common combinations of treatment agents in our database. Table 7 summarizes our findings. We found that 20.7% of participants took medications other than the 7 treatments we looked at. Vitamin C was the most common single agent used in the whole database, with 16.2% of the participants receiving it therapeutically. 12.2% reported receiving no treatment. The most common combination was azithromycin with vitamin C, with 10.2% of participants receiving it. We noted 7.9% of participants receiving vitamins C and D and 7.8% reporting they took azithromycin, vitamins C & D, and Zinc. Vitamin C was used in the vast majority of the combinations (8 out of 12), with azithromycin and vitamin D in second place (5/12 for each). Vitamins C and D were the most occurring pain in the combinations observed. The observed combinations accounted for more than 99% of the combinations observed in this study.

## 4. Discussion

The data about COVID-19 pandemic in Yemen is limited, and studies are challenged by the lack of diagnostic testing, where in a prior study, only 1.6% of populations with clinical diagnosis of COVID-19 infection had their diagnosis confirmed with a PCR test [6]. COVID-19 prescription patterns and rate of medication and/or supplement intake has not been well described in medical literature. Although this is a questionnaire based observational study, our findings provide some interesting insights about prescription patterns, by demographics (sex and age groups) and by disease severity (outpatient, hospitalized, and with respiratory failure). It also looks at the trends of medications and supplements prescribing, and the most common combinations of medications used in a resource limited country.

Our study is unique in trying to shed light on the treatment disparities among sexes and age groups, especially in a war-torn country in the middle east in the setting of a pandemic. In the case of COVID-19 infection, it was clear early on the increased age was a risk factor and predictor of poor outcome in COVID-19 infection [7–9]. That is possibly the reason why we have noted a steady increase of the likelihood of receiving treatment with older age groups. As for the disparities in treatment among sexes, the data has been mixed. A meta-analysis from 2016 showed that Women were 27% more likely than men to receive an antibiotic prescription[10]. Pain management, on the other hand, seemed to have significant gender bias against women [11], despite the clear data that shows women pain is physiologically more pronounced than men [12].

This study was conducted in May of 2020, when there were several attempts to find effective treatment for the COVID-19 infection. At that point, the data on using agents such as hydroxychloroquine, azithromycin, vitamin D, and others to help treat the infection and decrease the severity of the illness has failed to demonstrate a statistically significant change. The literature on prescription and usage patterns for agents other than antibiotics in COVID-19 infection remains scarce. Our study also looked at systemic corticosteroids treatment months prior to the RECOVERY trial was published, at a time when corticosteroids usage was still controversial [13].

In accordance with the global trend, the use of hydroxychloroquine and azithromycin was remarkable in our study population. During the time this survey was conducted, the use of Hydroxychloroquine was a common practice for treating COVID-19 infection as well as for prophylaxis around the world. At the same time, more data was coming out about the lack of efficacy. Eventually, both medications were not shown to be effective in COVID-19 treatment [14]. Around one third of the respondents who were treated as outpatients received antibiotics, a rate similar to previously published studies [15]. Our study also highlighted the increased tendency to prescribe antimicrobial to hospitalized patients, where two thirds of the patients received antibiotics, which has been similar to what has been reported in literature (72%–90% [16, 17]).

Out of the vitamins and minerals we looked at in our study, vitamin C was the most commonly used supplement in this study, with 62% receiving it alone or as part of their treatment regimen. This might be due to the historical trend of consuming vitamin C in cases of influenza and upper respiratory tract infection. Vitamin D came second, with 32.8% of respondents receiving it as therapy. This study predates the body of literature that signaled a worse outcome with vitamin D deficiency and COVID-19 infection [18]. Zinc came third, with 21.5% of the respondents being treated with. Indirect observational evidence suggests zinc may potentially reduce the risk, duration and severity of COVID-19 infections, especially in cases of zinc deficiency [19].

Finally, the rate of using two anti-inflammatory agents’ treatments: paracetamol (acetaminophen) and systemic steroids was explored in this study. The utilization of paracetamol was surprisingly low (8% total, with decreased frequency of usage with increased clinical severity). This is probably due to the initial warnings of the potential harmful effects of using anti-inflammatory medications in COVID-19 infections at the beginning of the pandemic [20]. That previous insight probably contributed to the low utilization of systemic steroids as well (1.5%, 2% for admitted patients), especially before the results of the RECOVERY trial were published in July of 2020. This study has several limitations: one of these is being an online questionnaire-based study. It also did not include diagnostic tests such as real-time polymerase chain reaction or lung computed tomography results.

## 5. Conclusion

This study provides some important information about the commonly observed treatments and prescription patterns during the COVID-19 pandemic in Yemen during May- July of 2020. Vitamin C was the most prescribed treatment and was used in the vast majority of the combinations, with azithromycin and vitamin D in second place. Around one third of the respondents who were treated as outpatients received antibiotics. The study reflects the influence of global trends in medication prescription in resource-limited countries.

## Figures and Tables

**Table 1:** General Demographic Characteristics of the respondents

Demographic Characteristic	*n*	%
Male gender	1,210	51.70%
Age group (Mode)	21–30	31.90%
Chronic diseases	595	25.40%
Diabetes Mellitus	151	6.50%
Hypertension	180	7.70%
Obesity	233	10.00%
Asthma	54	2.30%
Hospitalized	147	6.30%
Developed respiratory failure requiring supplemental O2 or mechanical ventilation	92	3.90%

**Table 2:** Prescription pattern by age group and gender

	Medication count (%)	No meds count (%)	Total
**Total**	2087 (89.1%)	254 (10.9%)	2341
**Female**	977 (86.4%)	154 (13.6%)	1131
**Male**	1110 (91.7%)	100 (8.3%)	1210
**1 Day - 10 Years**	71 (74.7%)	24 (25.3%)	95
**11 – 20 Years**	199 (74.8%)	67 (25.2%)	266
**21 – 30 Years**	684 (91.7%)	62 (8.3%)	746
**31 – 40 Years**	590 (91.9%)	52 (8.1%)	642
**41 – 50 Years**	284 (92.8%)	22 (7.2%)	306
**51 – 60 Years**	149 (90.3%)	16 (9.7%)	165
**61 – 70 Years**	74 (89.2%)	9 (10.8%)	83
**71 Years or older**	36 (94.7%)	2 (5.3%)	38

**Table 3:** Respondents by city name and ratio to governate

Governorate	Population (as per 2019 population estimation)	Number of respondents (% of sample)	Sample per 100,000 of the population
Abyan	513,701	7 (0.30%)	1.36
Ad Dali	602,613	6 (0.26%)	1
Aden	1,087,653	146 (6.24%)	13.42
Al Bayda	835,683	8 (0.34%)	0.96
Al Hudaydah	3,774,914	106 (4.53%)	2.81
Al Jawf	663,147	4 (0.17%)	0.6
Al Mahwit	732,360	23 (0.98%)	3.14
Amanat Al Asimah (Capital municipality)	1,174,767	1349 (52.6%)	114.83
Amran	1,123,651	49 (2.09%)	4.36
Dhamar	1,697,067	147 (6.28%)	8.66
Hadhramaut	1,329,085	6 (0.26%)	0.45
Hajjah	1,887,213	43 (1.84%)	2.28
Ibb	3,911,070	208 (8.89%)	5.32
Lahij	926,291	10 (0.43%)	1.08
Ma’rib	504,696	27 (1.15%)	5.35
Sa’dah	987,663	11 (0.47%)	1.11
Sana’a Governorate	2,279,665	93 (3.97%)	4.08
Ta’izz	4,554,443	98 (4.19%)	2.15
**Total**	**28,585,682**	**2341 (100%)**	**8.19**

**Table 4:** List of medications, supplements, herbs and food that was reported as part of a regimen to treat COVID-19 infections

Medications	Supplements	Herbs and food
Aspirin	Multivitamins	Honey
Guaifenesin	Vitamin B complex	Black seed
Solpadine (Paracetamol + Codeine)	Vitamin B12	Lemon
Cefaclor	Selenium	Lime
Amoxicillin	Omega 3	Clove
Ceftriaxone		Costus
Levofloxacin		Ginger
Ciprofloxacin		Roselle
Antibiotic capsules		Onions
Fraxiparine		garlic
Enoxaparin		Fruits
esomeprazole		Vegetables

**Table 5:** Frequency of medication prescribing by hospitalization status and respiratory failure

Medication	All	Outpatient (%)	Hospitalized (%)	Respiratory failure (%)
**Hydroxychloroquine**	140 (5.9%)	117 (5.3%)	23 (15.6%)	19 (24.7%)
**Azithromycin**	788 (33.7%)	704 (32.1%)	84 (57.1%)	52 (67.5%)
**Vit C**	1451 (62%)	1,361 (62.0%)	90 (61.2%)	57 (74.0%)
**Vit D**	767 (32.8%)	712 (32.5%)	55 (37.4%)	38 (49.4%)
**Zinc**	504 (21.5%)	464 (21.1%)	40 (27.2%)	35 (45.5%)
**Paracetamol**	189 (8.0%)	187 (8.5%)	2 (1.4%)	3 (3.9%)
**Corticosteroids**	19 (0.8%)	13 (0.5%)	2 (1.4%)	4 (5.2%)
**Other antibiotics**	34 (1.5%)	31 (1.4%)	3 (2.0%)	2 (2.1%)

**Table 6:** Hydroxychloroquine prescribing before and after the diagnosis

**Was the patient prescribed Hydroxychloroquine after diagnosis**		**Was the patient on Hydroxychloroquine?**		
		**Yes (%→)**	**No (%→)**	**Total**
		**(%↓)**	**(%↓)**	
	**Yes**	18 (14.1%)	122 (87.1%)	140
		−12.90%	−5.50%	
	**No**	110 (5.0%)	2091 (95.0%)	2201
		−85.90%	(94.9%→)	
	**Total**	128	2213	2341

**Table 7:** Most common medication combinations prescribed

No Meds	Hydroxychloroquine	Azithromycin	Vit C	Vit D	Zinc	Paracetamol	Number of Records	% of all regimens
FALSE	FALSE	FALSE	FALSE	FALSE	FALSE	FALSE	431	20.7%
FALSE	FALSE	FALSE	TRUE	FALSE	FALSE	FALSE	338	16.2%
TRUE	FALSE	FALSE	FALSE	FALSE	FALSE	FALSE	254	12.2%
FALSE	FALSE	TRUE	TRUE	FALSE	FALSE	FALSE	213	10.2%
FALSE	FALSE	FALSE	TRUE	TRUE	FALSE	FALSE	165	7.9%
FALSE	FALSE	TRUE	TRUE	TRUE	TRUE	FALSE	163	7.8%
FALSE	FALSE	FALSE	TRUE	TRUE	TRUE	FALSE	146	7.0%
FALSE	FALSE	TRUE	TRUE	TRUE	FALSE	FALSE	112	5.4%
FALSE	FALSE	TRUE	FALSE	FALSE	FALSE	FALSE	95	4.6%
FALSE	FALSE	FALSE	FALSE	FALSE	FALSE	TRUE	64	3.1%
FALSE	TRUE	TRUE	TRUE	TRUE	TRUE	FALSE	54	2.6%
FALSE	FALSE	FALSE	TRUE	FALSE	FALSE	TRUE	48	2.3%
